# Effect of the Medicinal *Agaricus blazei* Murill-Based Mushroom Extract, AndoSan^TM^, on Symptoms, Fatigue and Quality of Life in Patients with Crohn’s Disease in a Randomized Single-Blinded Placebo Controlled Study

**DOI:** 10.1371/journal.pone.0159288

**Published:** 2016-07-14

**Authors:** Stig Palm Therkelsen, Geir Hetland, Torstein Lyberg, Idar Lygren, Egil Johnson

**Affiliations:** 1 Department of Gastrointestinal and Pediatric Surgery, Oslo University Hospital, Ullevål, Oslo, Norway; 2 Immunology and Transfusion Medicine, Oslo University Hospital, Ullevål, Oslo, Norway; 3 Medical Biochemistry, Oslo University Hospital, Ullevål, Oslo, Norway; 4 Medicine, Oslo University Hospital, Ullevål, Oslo, Norway; 5 Faculty of Medicine, University of Oslo, Oslo, Norway; University Hospital Llandough, UNITED KINGDOM

## Abstract

**Background:**

Ingestion of AndoSan^TM^, based on the mushroom *Agaricus blazei* Murill, has previously shown an anti-inflammatory effect through reduction of pro-inflammatory cytokines in healthy individuals and patients with Crohn’s disease (CD). In this randomized single-blinded placebo-controlled study we examined whether intake of AndoSan^TM^ also resulted in clinical effects.

**Methods and Findings:**

50 patients with symptomatic CD were randomized for oral daily consumption of AndoSan^TM^ or placebo for a 21-day experimental period, in this per-protocol study. Patients reported validated scores for symptoms, fatigue and health related quality of life (HRQoL) at days 0, 14 and 21. Fecal calprotectin and general blood parameters were also analyzed. In the AndoSan^TM^ group (n = 25) symptoms improved from baseline (day 0) to days 14 and 21, with respective mean scores (95% CI) of 5.52 (4.64–6.40), 4.48 (3.69–5.27) and 4.08 (3.22–4.94) (p<0,001). We found significant improvements in symptom score for both genders in the AndoSan^TM^ group, and no significant changes in the placebo (n = 25) group. There were however no significant differences between the groups (p = 0.106), although a marginal effect in symptom score for men (p = 0.054). There were comparable improvements in physical, mental and total fatigue for both groups. HRQoL versus baseline were at day 21 improved for bodily pain and vitality in the AndoSan^TM^ group and for vitality and social functioning in the placebo group. No crucial changes in general blood samples and fecal calprotectin were detected.

**Conclusions:**

The results from this single-blinded randomized clinical trial shows significant improvement on symptoms, for both genders, in the AndoSan^TM^ group, but no significant differences between the study groups. The results on fatigue, HRQoL, fecal calprotectin and blood samples were quite similar compared with placebo. The patients did not report any harms or unintended effects of AndoSan^TM^. CD patients with mild to moderate symptoms may have beneficiary effects of AndoSan^TM^ as a safe supplement in addition to conventional medication.

**Trial Registration:**

ClinicalTrials.gov NCT01496053

## 1. Introduction

A mushroom *Agaricus blazei* Murill (AbM) has for centuries been utilized as a health food ingredient by the local population in the Piedade area in Brazil, where prevalence of atherosclerosis, hyperlipidemia, diabetes and cancer was lower than in neighboring regions [1], presumably owing to AbM consumption. In 1966 the mushroom was brought to Japan and introduced to the health food market. Since then, AbM and other *Basidiomycetes* mushrooms [2, 3] have been subjected to an increasing research effort regarding their effects.

AbM *per se* and the AbM-based mushroom extract, AndoSan^TM^ (ACE Co. Ltd., Gifu-ken, Japan), composed of AbM (82.4%), *Hericium erinaceus* (He) (14.7%) [2] and *Grifola frondosa* (Gf) (2.9%) [3], contain immunomodulatory ß-glucans and other biologically active substances like α-glucans [4], proteoglucans [5], lectins [6], ergosterol (provitamin D2) [7], agaritine [8], isoflavonoids [9], anti-oxidant [10], and anti-inflammatory substances [11] including the 4-HM steroid [12].

Depending on the experimental set up, AbM or the AbM-based extract AndoSan^TM^, comprise as reviewed [13, 14] anti-tumor, anti-allergic, and anti-inflammatory effects *in vivo*.

AndoSan^TM^ is an extract of the mushrooms´ mycelium and not their fruiting bodies and was recently shown to contain less ß-glucan [15] than anticipated from the published data on AbM fruiting body. Therefore, action also of other yet not identified immunomodulating substances in this particular extract must part-take to render the observed effects. An example is an isolated fraction of AndoSan^TM^ that was found to inhibit the production in macrophages of the tumor-associated and pro-inflammatory protease, legumain [15].

In patients with Crohn’s disease (CD) increased mucosal levels have been demonstrated for MIP-1ß, MCP-1 and IL-8 [16], IL-1ß [17], IL-6 and TNFα [18]. Cytokine levels in serum are less well studied but increased levels have been reported for IL-6 [18] and TNFα [19, 20]. Moreover, in a recent extensive review [21] the cytokines IFNγ, IL-6, IL-7 and IL-8 were considered to be persistently elevated in blood of CD patients compared with findings in healthy individuals.

In 11 patients with CD who consumed the mushroom extract AndoSan^TM^ for 12 days [22] cytokine levels were reduced in untreated (IL-2, IL-8, IL-17) and in LPS-stimulated blood *ex vivo* (IL-1ß, MIP-1ß, MCP-1, IL-8, IL-17, G-CSF and GM-CSF). Then, the next step was to examine whether a decline in pathological levels of cytokines mediated by the mushroom extract *in vivo*, did result in a putative beneficial clinical effect in patients with CD.

We have in a recent single-blinded randomized placebo controlled study, in which patients with ulcerative colitis received this mushroom extract (AndoSan^TM^) for three weeks [23], demonstrated improvements in symptoms, fatigue and HRQoL compared with patients in the placebo group.

On this background, it was pertinent to study whether consumption of AndoSan^TM^ had similar effects in patients with CD.

## 2. Materials and Methods

### 2.1. Reagents

The mushroom extract AndoSan^TM^ was provided by the company Immunopharma AS (organization no. 994924273), Oslo, Norway. It was stored at 4°C in metal cans and used under sterile conditions *ex vivo* and kept sterile until taken by volunteers for *in vivo* experiments. This mushroom extract is a commercial product produced by the company ACE Co. Ltd., Gifu-ken, Japan, for Immunopharma AS. The AbM mixed powder contains per 100 g the following constituents: moisture 5.8 g, protein 2.6 g, fat 0.3 g, carbohydrates 89.4 g, of which ß-glucan constitutes 2.8 g, and ash 1.9 g. The AndoSan^TM^ extract contains 82.4% of *Basidiomycetes* mushroom derived from AbM, 14.7% from He [2] and 2.9% from Gf [3], and its final concentration was 340 g ⁄ l. The amount per litre of the extract was for sodium 11 mg, phosphorus 254 mg, calcium 35 mg, potassium 483 mg, magnesium 99 mg and zinc 60 mg. The LPS (lipopolysaccharide) content of AndoSan^TM^ was found, using the Limulus amebocyte lysate test (COAMATIC Chromo-LAL; Chromogenix, Falmouth, MA, USA) with detection limit 0.005 EU ⁄ ml (1 EU = 0.1 ng ⁄ ml), to be a miniscule concentration of <0.5 pg ⁄ ml. AndoSan^TM^ had been heat-sterilized (124°C for 1 h) by the producer. Several tests from Japan Food Research Laboratories (authorized by the Japanese Government) were done in March 2012, December 2013, October 2014, April 2015 and February 2016. The tests were for pH, arsenic, lead, cadmium, tin, aerobic plate count, coliform bacteria (MPN), viable molds count, viable yeasts count, mesophilic aerobic spores, refractometric brix degree and specific gravity (15°C)–and all of the results were within the quantitation limits. AndoSan^TM^ also passed the water quality test (no bacteria, acceptable level of ions, pH, taste, color and odor). An accelerated aging test (up to four months) with almost unchanged character of the mushroom drink. AndoSan^TM^ were also tested for radioactivity, with no detection of Cesium-137, Cesium-134 and Iodine-131 (Meiji Co, Japan). In addition, the Norwegian Food Safety Authorities found no radioactivity.

### 2.2. Analyses

Blood was harvested from the antecubital vein into glass tubes containing 15 IU heparin per ml or 10 mmol EDTA per ml. The EDTA blood was each time (days 0, 14 and 21) analyzed for haemoglobin, haematocrite, mean cellular volume, mean cellular haemoglobin, reticulocytes, immature reticulocytes, leukocytes including a differential count of neutrophils, basophils, eosinophils, lymphocytes and monocytes, thrombocytes, C-reactive protein (CRP), urea, creatinine, bilirubin, aspartate aminotransferase, alanine aminotransferase, lactate dehydrogenase, γ-glutamine transferase, alkaline phosphatase and pancreatic amylase. Fecal calprotectin concentrations (mg/kg) (normal value <50 mg/kg) at days 0, 14 and 21 were determined in duplicates as reported [24, 25].

The patient-reported symptom score was the simple Crohn’s disease Activity Index (SCDAI) also denounced the Harvey-Bradshaw index [26]. The simple index is based on five graded items; general well-being (very well = 0, slightly below par = 1, poor = 2, very poor = 3, terrible = 4), abdominal pain (none = 0, mild = 1, moderate = 2, severe = 3), number of liquid stools per day (1 = 0, 2 = 1, 3–4 = 2, 5–6 = 3, 7–9 = 4, > 9 = 5), abdominal mass (this item was not examined) and extraintestinal manifestations (arthralgia, uveitis, erythema nodosum, aphthous ulcers, pyoderma gangrenosum, anal fissure, new fistula, abscess (score 1 per item)). The symptom score ranges from 0–21. Scores 3–5 meant mild, 6–9 moderate and over 9 severe disease activity. A criterion for inclusion was a score beyond 2.

Self reported health-related quality of life (HRQoL) was assessed with the short form 36 (IQOLA SF-36 Norwegian version 1.2), which is a generic HRQoL questionnaire consisting of 36 items, of which 35 are grouped into the following eight health domains: (1) physical functioning (PF), (2) social functioning (SF), (3) role limitations due to physical problems (RP), (4) role limitation due to emotional problems (RE), (5) mental health (MH), (6) vitality (VT), (7) bodily pain (BP) and (8) general health perception (GH). Each domain is graded on a scale of 0–100, and the higher the score the better the HRQoL. The validity and reliability of the SF-36 form have been demonstrated for a number of countries including Norway (version 1) [27]. The data were compared with published norms from 2323 individuals in the general population. Only 30 out of 5400 HRQoL questions were unanswered, and accordingly, 17 out of 1200 dimensions were lacking. Using a scoring algorithm for missing data outlined in the SF 36 survey manual, still 5 out of 17 dimensions involving 3 patients in the placebo group, could not be included in the results.

Fatigue consists of total fatigue (11 items of graded questions with score 0–3 per question), which is the sum of physical fatigue (7 items) and mental fatigue (4 items), which has been validated in a Norwegian general population [28]. The respective scores for total, mental and physical fatigue are 0–33, 0–21 and 0–12, and the higher score the more fatigue. The items of physical (1–7) and mental (8–11) fatigue were: 1) Do you have problems with tiredness? 2) Do you need to rest more? 3) Do you feel sleepy or drowsy? 4) Do you have problems with starting things? 5) Are you lacking in energy? 6) Do you have less strength in your muscles? 7) Do you feel weak? 8) Do you have difficulty concentrating? 9) Do you have problems thinking clearly? 10) Do you make slips of the tongue when speaking? 11) How is your memory? Criteria for chronic fatigue syndrome was a dichotomized score >4 and duration>6 months.

### 2.3. Inclusion of Patients

**Fig 1:**
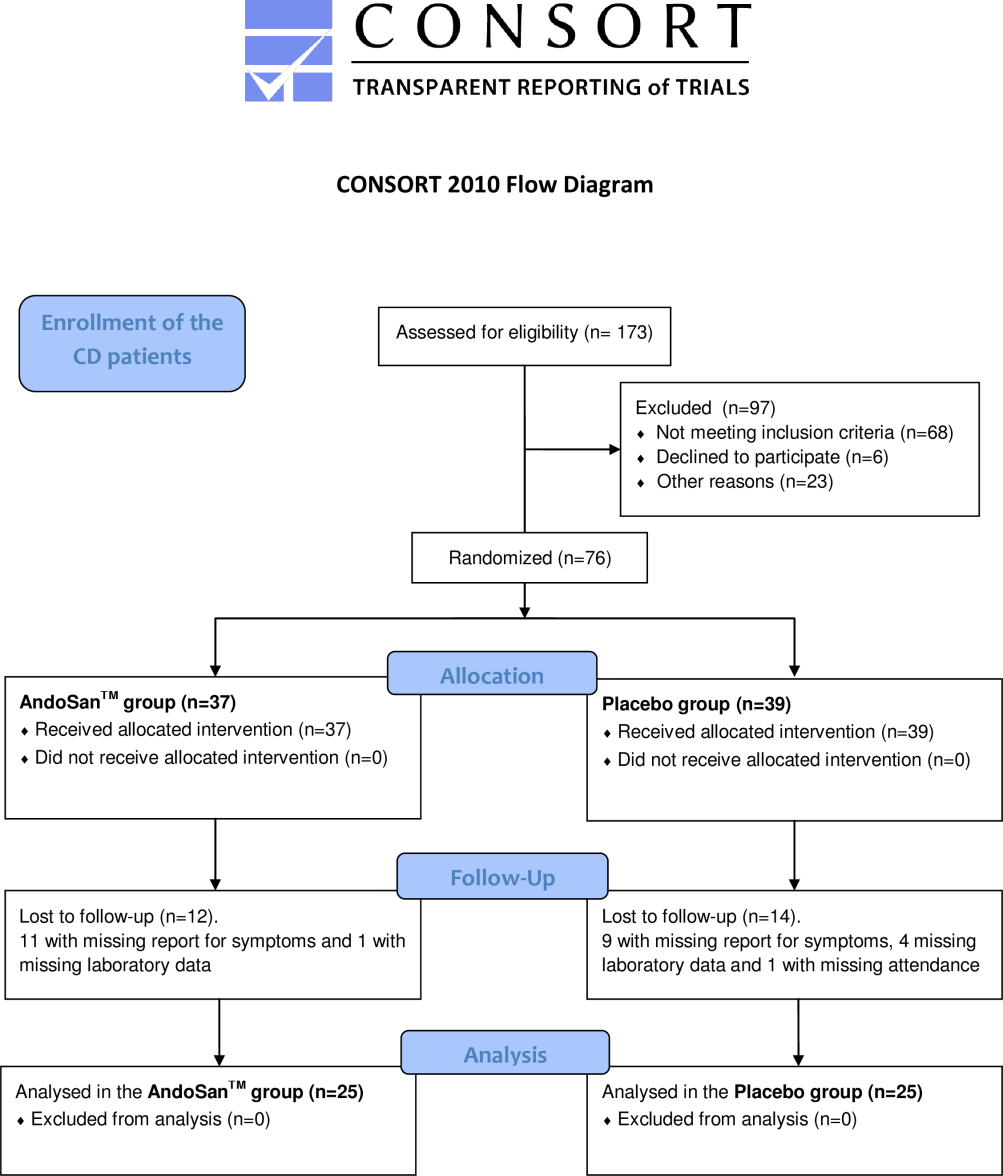
An algorithm showing the scheme for inclusion of the patients in the study.

173 patients with CD were phone interviewed and those with SCDAI score of at least 3 were given the opportunity to join the study. At the first attendance SCDAI was re-recorded and criteria for exclusion were pregnancy, biological treatment with antibodies to TNFα (Adalimumab, Infliximab), daily use of more than 5 mg of prednisolone, change of medication and/or consumption of mushroom products from two weeks before till end of the study. A flow chart reveals additional reasons for exclusions (Fig 1). The 23 excluded patients in the initial screening mentioned as “other reasons” were: 10 could not participate because of not able to attend, 5 had moved to a different part of the country, 3 never showed up, and 1 proved to be pregnant, and one each had an ileostomy with high output, drug abuse, severe comorbidity or language difficulties.

### 2.4. Experimental Design and Randomization

This is a single-center randomized two-armed patient-blinded study designed to determine whether daily oral intake of a mushroom extract, AndoSan^TM^, improved clinical symptoms, fatigue and quality of life in patients with CD during the 21 days’ study period. The patients were evaluated before (visit 1, day 0), during (visit 2, day 14) and after (visit 3, day 21) consumption of AndoSan^TM^ (30 ml twice daily). This dose (60 ml/day) reduced levels of pro-inflammatory cytokines and chemokines in healthy volunteers [24] and in patients with UC and CD [34], whilst half dosage (30 ml/day) had no detectable effects (unpublished data). The placebo group was evaluated likewise but received an equal volume of color-like drink with ionized water containing 0.5 ml per litre of caramel color (E150c) with salt.

Block-randomization was done after the phone interview, with uneven and even numbers given for AndoSan^TM^ or placebo, respectively. The patients, one by one, were placed in one pile, and the group affiliations were placed in another pile. The randomization was performed by combining one selection from each pile, both anonymized. The first author performed the randomization, enrolled the participants, and assigned participants to interventions. A few patients were excluded throughout the study by not attending or because of intercurrent incidents (disease, unexpected life events). Accordingly, a slight imbalance of the study groups occurred that was corrected for in the latter rounds of randomization. More specifically, the 50 CD patients were divided into 13 groups (range 1–9 per group), each with a study period of 3 weeks. The included 50 symptomatic patients had almost no missing data (only for 3 patients with missing a total of 5 out of 1200 dimensions for the SF-36 results) and were randomized and blinded for oral daily consumption (30 ml twice daily) of AndoSan^TM^ or placebo for the 21 days’ experimental period. Patients in the AndoSan^TM^ group and the placebo group self-reported, in written, at visit 1 (day 0), visit 2 (day 14) and visit 3 (day 21) regarding symptoms, fatigue and health-related quality of life. Patient derived blood samples and fecal calprotectin from these visits were also analyzed. All data were stored in a secure database (Access–Microsoft Office) at a server at Oslo University Hospital, Ullevål, Norway. A study number anonymized the patients.

This study on clinical outcome is a follow up of a previous pilot study [22] in which there was a reduction of pro-inflammatory cytokines and chemokines in patients receiving the same daily dose of AndoSan^TM^, but for 12 days. Prospective differences of 20% between the experimental and placebo group and assumed standard deviation of 20% for the different parameters with a significant level of 5% and a power of 90% (ß = 0.10), demands about 25 patients per randomized arm (calculated in cooperation Oslo Center for Biostatistics and Epidemiology, Oslo University Hospital).

### 2.5. Patient Characteristics

Disease duration for the CD patients was 9.7 years (range 0.5–46) and 8.0 years (range 0.5–42) in the AndoSan^TM^ and placebo groups, respectively. Disease location and behavior, number and type of resections as well as proportions of patients subjected to surgery were registered (Table 1). There were 30 and 35 extra-intestinal manifestations in 21 patients in the AndoSan^TM^ group and 22 patients in the placebo group, respectively. Comorbidities, exclusively in the AndoSan^TM^ group, were Mb Bechterew in 3, diabetes mellitus in 1 and chronic obstructive lung disease in 1 patient(s). Combinations of 5-ASA, azathioprine and budesonide or prednisolone were consumed in 3 and 2 patients, respectively. Topical treatment with 5-ASA was used in 3 patients in the AndoSan^TM^ group.

**Table 1:** Demographic and patient data.

	AndoSan^TM^	Placebo	P
**Number**	25	25	1.00
**Age** (years)	41.0 (25–74)	42.0 (22–70)	0.59
**Gender** (male, female)	11, 14	10, 15	0.78
**Duration of diagnosis** (years)	9.7 (0.5–46)	8.0 (0.5–42)	0.68
**Disease location**			
L1 terminal ileum	11	8	0.38
L2 colon	5	5	1.00
L3 ileocolon	9	12	0.39
**Behavior**			
B1 inflammatory	12	19	0.04
B2 stricturing	12	5	0.04
B3 perforating	0	1	0.31
**Resection**			
Ileal	6	0	0.01
Ileocolic	13	5	0.02
Colic	1	4	0.16
Total (number of resections (patients))	20 (9)	9 (6)	0.002 (0.35)
**Medication during the study period**			
None	7	11	0.24
5-ASA/Azathioprin	9	8	0.77
Low dose of steroids	4	4	1.00
Several medications	3	2	0.64
Topical treatment	3	0	0.07
**Extra-intestinal manifestations**			
None	4	3	0.68
Arthralgia	17	17	1.00
Anal fissures	7	7	1.00
Aphtous ulcers	3	6	0.27
Erythema nodosum	2	2	1.00
Anal fistula	0	2	0.15
Pyoderma gangrenosum	1	0	0.32
Enteric fistula	1	0	0.32

Values for age and duration of diagnosis are given as median (range). P values between groups is measured with unadjusted chi square test, and Mann-Whitney U test for age and gender.

### 2.6. Statistical Analysis

Data are presented as mean and standard deviation or as median and range values. Paired sample t-test and Wilcoxon test were used for within-group analysis. The judgment of whether the distributions of the main efficacy variables were so close to the normal distribution that normality-based significance tests may be used, also for each individual index at baseline that compose the SCDAI, is based on the finding in a paper by Fagerland and Sandvik [29]. Mixed models corrected for baseline values were used for measuring P values between the AndoSan^TM^ and placebo groups, using V1, V2 and V3 with time as a continuous variable. P values below 0.05 were considered statistically significant. The SPSS statistical program for the social sciences, version 22 (IBM) was used in the analyses.

### 2.7. Ethical Considerations

The study was approved on April 8, 2011, by the regional ethics committee (REC–South East Norway, ref. 2011/404) and followed the guidelines of the Helsinki declaration. The participants were informed and signed a written consent for participation, including the option of study withdrawal. The patients were recruited and followed up at the Department of Medicine, Oslo University Hospital, Ullevål, Norway, in the period of June 2012 to May 2014. The study was registered with unique protocol ID AbM2012-IBD and clinical trials gov ID NCT 01496053 (December 15, 2011). The authors confirm that all ongoing and related trials for this drug/intervention are registered.

## 3. Results

### 3.1. Exclusion of randomized patients

A total of 76 patients, 37 in the AndoSan^TM^ group and 39 in the placebo group, were randomized for inclusion in this study. 26 of these patients were excluded according to the criteria of the study protocol, because of missing data on symptom score, laboratory data and not attending in 20, 4 and 1, patient(s), respectively. Thereby we ended up with 25 patients in the AndoSan^TM^ group and 25 in the placebo group (Fig 1).

### 3.2. Age and Gender

Median age for the 50 included patients with CD was 41 years (range 22–74). There were 11 men and 14 women in the AndoSan^TM^ group and 10 men and 15 women in the placebo group. Respective ages in the two groups were median 42 (range 22–70) and 44.5 (28–74) for men (p = 0.611) and 43 (26–69) and 38 (25–61) for women (p = 0.36).

### 3.3. Symptom Score

The symptom scores were similar at inclusion in the AndoSan^TM^ and placebo groups, with respective mean scores of 5.52 and 5.04. There were no significant differences in baseline symptom scores between male and females within the two groups. Compared with baseline there were in the AndoSan^TM^ group increasing reductions of symptom score for both genders from visit 2 (day 14) to visit 3 (day 21) (Table 2). In the placebo group, only for women there was a reduction of symptom score from baseline to visit 2 but not visit 3. When comparing the two groups using mixed models corrected for baseline values for both genders there was no difference (p = 0.106). However, for men, there was a close to significant difference (p = 0.054) in favor of the mushroom group.

**Table 2:** Symptom score (SCDAI) for the CD patients.

	V1	V2	V3	P_V1V2_	P_V1V3_	P_between groups_
**AndoSan**^**TM**^	5.52 (4.64–6.40)	4.48 (3.69–5.27)	4.08 (3.22–4.94)	0.001	<0.001	0.106
M (n = 11)	5.05 (4.07–6.11)	3.64 (2.72–4.55)	3.27 (1.99–4.55)	0.014	0.011	
F (n = 14)	5.85 (4.41–7.30)	5.14 (3.95–6.34)	4.71 (3.52–5.90)	0.035	0.014	
**Placebo**	5.04 (4.49–5.59)	4.52 (3.72–5.32)	4.68 (3.92–5.44)	0.119	0.327	
M (n = 10)	4.70 (4.11–5.29)	4.50 (2.74–6.26)	4.80 (3.23–6.37)	0.764	0.885	
F (n = 15)	5.27 (4.37–6.17)	4.53 (3.65–5.42)	4.53 (3.62–5.44)	0.044	0.085	

V1; visit 1 (day 0), V2; visit 2 (day 14), V3; visit 3 (day 21).

Values are given as means and 95% confidence intervals. Paired sampled t-test for the p-values.

P between groups is measured with mixed models corrected for baseline values.

Within the AndoSan^TM^ group there was for both genders a significant improvement in stool frequency with scores 2.04, 1.56 (p = 0.01) and 1.20 (p<0.01) at visit 1–3, respectively. There also was a trend for both genders towards reduction of abdominal pain with scores 1.12 and 0.88 (p = 0.06) at visit 1 vs. 3, with significant values for men (p = 0.04). When using mixed models corrected for baseline values there was a significant difference between the study groups for stool frequency (p = 0.011), in favor of the AndoSan^TM^ group, but not for abdominal pain (p = 0.36).

### 3.4. Fatigue Score

Firstly, the normative fatigue scores in the Norwegian population were compared with scores in the CD patients at inclusion in this study (Table 3). There were for both genders significant decreases of physical, mental and total fatigue scores in the CD patients compared with the general population. This effect was much more pronounced for physical fatigue than for mental fatigue.

**Table 3:** Mean fatigue scale scores. Normative data in the Norwegian population compared with the included CD patients.

	Normative data		CD		P_Normative data vs UC_	
	M (n = 1112)	F (n = 1175)	M (n = 21)	F (n = 29)	M	F
**Total**	11.9	12.6	16.43	17.76	<0.0001	<0.0001
	(3.9)	(4.0)	(4.48)	(5.87)		
**Physical**	7.6	8.2	11.38	12.14	<0.0001	<0.0001
	(3.0)	(3.2)	(3.69)	(4.14)		
**Mental**	4.3	4.4	5.05	5.62	0.0152	0.0052
	(1.4)	(1.4)	(1.40)	(2.16)		

Normative data from the general Norwegian population, age 19–80.

Values are given as mean and standard deviation (SD). Independent sample t-test for the p-values.

Twenty of the 50 patients (40%) had chronic fatigue at visit 1 vs. about 11% in the normative population [28]. The scores for genders on inclusion were quite similar within and between the groups (data not shown). In the AndoSan^TM^ group (Table 4) for both genders the CD patients reported a significant decline in total and physical fatigue, but not mental fatigue, from baseline to visit 3 (day 21). Mental fatigue was transiently improved at visit 2 but not at visit 3. In the placebo group, however, the three aspects of fatigue was improved both at visit 2 and 3.

**Table 4:** Fatigue scores for the patients with (n = 25 AndoSan^TM^ and n = 25 placebo) CD.

	AndoSan^TM^ group					Placebo group					
	V1	V2	V3	P_V1V2_	P_V1V3_	V1	V2	V3	P_V1V2_	P_V1V3_	P_Between groups_
**TF**	16.40	15.28	14.00	0.128	0.032	18.00	16.28	15.36	0.011	0.011	0.813
	(5.07)	(4.86)	(5.10)			(5.55)	(4.97)	(4.65)			
**PhF**	11.08	10.48	9.04	0.289	0.016	12.56	11.32	10.64	0.029	0.016	0.187
	(3.51)	(3.74)	(3.66)			(4.26)	(4.00)	(4.01)			
**MF**	5.32	4.80	4.96	0.045	0.265	5.44	4.96	4.72	0.008	0.031	0.824
	(1.97)	(1.56)	(1.77)			(1.83)	(1.43)	(1.10)			

V1; visit 1 (day 0), V2; visit 2 (day 14), V3; visit 3 (day 21).

TF; total fatigue, PhF; physical fatigue, MF; mental fatigue.

Values are given as mean and standard deviation (SD). Paired sampled t-test for p-values.

P between groups is measured with mixed models corrected for baseline values.

Moreover, when comparing the AndoSan^TM^ and placebo groups using mixed models corrected for baseline values there were no differences between the two groups regarding the three fatigue scores.

### 3.5. Quality of Life

HRQoL scores (SF-36) for CD patients were compared with age-adjusted normative data for the Norwegian population (Table 5). Men had significant reduction in scores of all 8 dimensions whilst women had similar results with the exception of physical functioning (PF), which was within the normal range. The reductions of quality life scores in the CD patients were for both genders most pronounced for the dimensions vitality (VT), general health (GH) and role limitations, physical (RP) (p values < 0.0001) as compared with the general population.

**Table 5:** Mean SF-36 scale scores. Age-adjusted Normative Data from the Norwegian Population compared with patients with CD on inclusion.

	Normative data		Crohn´s disease		P_Normative data vs CD_	
	M (n = 977–1017)	F (n = 1013–67)	M (n = 21)	F (n = 29)	M	F
**PF**	91.37 (16.19)	87.72 (17.53)	83.97 (15.33)	84.48 (15.49)	0.0383	0.3250
**RP**	83.27 (31.98)	79.18 (34.99)	51.19 (42.92)	31.90 (35.92)	0.0028	<0.0001
**BP**	78.06 (24.61)	74.46 (26.00)	58.86 (21.36)	55.07 (20.04)	0.0004	<0.0001
**GH**	78.34 (20.98)	77.46 (22.14)	46.19 (23.70)	46.98 (21.92)	<0.0001	<0.0001
**VT**	63.36 (18.19)	57.57 (21.00)	34.52 (21.56)	32.26 (14.74)	<0.0001	<0.0001
**SF**	88.23 (20.52)	84.78 (22.79)	68.45 (27.28)	62.05 (22.94)	0.0036	<0.0001
**RE**	85.89 (28.47)	80.94 (33.10)	66.67 (38.99)	65.52 (43.17)	0.0361	0.0144
**MH**	79.74 (15.75)	77.64 (16.85)	71.24 (17.46)	65.57 (19.30)	0.0148	0.0002

Age-adjusted normative data from the general Norwegian population, age 19–69.

Values are given as mean and standard deviation (SD). Independent sample t-test for the p-values.

SF-36; Short form 36, PF; physical functioning, RP; role limitations, physical, BP; bodily pain, GH; general heath perception, VT; Vitality, SF; social functioning, RE; role limitations, emotional, MH; mental health.

In the AndoSan^TM^ group as a whole the HRQoL score were at visit 3 significantly improved (Table 6) for bodily pain (BP) and VT, whilst VT and SF were improved in the placebo group.

**Table 6:** Mean SF-36 scale scores (n = 25 AndoSan^TM^ and n = 25 placebo) CD.

	AndoSan^TM^ group					Placebo group					
	V1	V2	V3	P_V1V2_	P_V1V3_	V1	V2	V3	P_V1V2_	P_V1V3_	P_AvsP_
**PF**	85.80	84.60	83.80	0.417	0.470	82.73	85.89	87.20	0.126	0.072	0.083
	(14.8)	(14.4)	(14.7)			(15.9)	(13.7)	(14.4)			
**RP**	41.00	54.00	49.00	0.040	0.088	39.00	39.00	42.00	1.000	0.671	0.120
	(43.2)	(39.3)	(44.2)			(36.9)	(37.6)	(40.7)			
**BP**	53.96	58.88	63.16	0.108	0.028	59.54	58.50	60.71	0.805	0.764	0.292
	(20.3)	(18.6)	(22.0)			(20.8)	(17.3)	(18.0)			
**GH**	50.64	48.52	51.08	0.396	0.886	42.66	45.08	46.32	0.328	0.156	0.454
	(23.4)	(23.6)	(26.1)			(21.1)	(19.5)	(18.5)			
**VT**	32.53	40.00	39.20	0.008	0.045	33.96	36.32	42.92	0.400	0.019	0.777
	(20.0)	(21.5)	(22.9)			(15.6)	(17.8)	(18.7)			
**SF**	65.50	74.00	72.00	0.021	0.152	64.06	66.67	72.40	0.447	0.032	0.537
	(25.8)	(20.7)	(21.1)			(24.3)	(22.3)	(24.7)			
**RE**	69.33	65.33	73.33	0.372	0.503	62.50	56.94	58.33	0.405	0.588	0.358
	(41.9)	(41.4)	(40.8)			(40.9)	(43.4)	(42.0)			
**MH**	69.28	71.04	69.76	0.440	0.836	66.67	71.00	71.00	0.057	0.164	0.392
	(17.1)	(13.3)	(18.0)			(20.3)	(17.5)	(19.0)			

Paired sampled t-test for the p-values.

P between groups is measured with mixed models corrected for baseline values.

When broken into gender the respective improvements at visit 3 were BP for men and SF for women in the AndoSan^TM^ group versus none in the placebo group (data not shown). There were no significant differences when comparing the two groups, using mixed models corrected for baseline values.

### 3.6. Calprotectin in Feces and Effect on General Blood Parameters

The patients delivered fecal tests at visits 1, 2 and 3. In the AndoSan^TM^ group (n = 25) the median (range) values for fecal calprotectin (mg/kg) were 394 (17–6000), 398 (20–2244) and 472 (36–1623), respectively. In the placebo group the corresponding values were 293 (21–2783), 515 (12–6000) and 342 (10–4659). There were no significant differences in levels of calprotectin within or between the groups, also when broken into gender (data not shown).

The following blood samples were analyzed at visit 1and 3: CRP, leukocytes, eosinophils, basophils, neutrophils, lymphocytes, monocytes, hemoglobin, haematocrite, mean cellular volume, mean cellular haemoglobin, immature reticulocytes, reticulocytes, thrombocytes, urea, creatinine, and GFR (glomerular filtration rate), bilirubin, aspartate aminotransferase, alanine aminotransferase, lactate dehydrogenase, γ-glutamine transferase, alkaline phosphatase and pancreatic amylase. Significant changes were for reduction of bilirubin (μmol/L) from 11.4 to 9.2 (p = 0.02) in the AndoSan^TM^ group and for increase of thrombocytes (10^9^/L) from 289 to 302 (p = 0.03) in the placebo group.

The median and range of blood samples of special interest were haemoglobin (g/l), leukocyte counts (10^9^/l) and CRP levels for visit 1 and 3 the AndoSan^TM^ group (n = 25) were 13.4 (12.0–16.2) versus 13.3 (11.4–16.0), 6.1 (3.1–12.2) versus 6.8 (2.7–13.0) and 2.9 (0.6–30.9) versus 2.1 (0.6–25.3), respectively. Corresponding values in the placebo group (n = 25) were 13.7 (10.5–15.5) versus 13.6 (10.7–15.5) for haemoglobin, 6.6 (4.2–13.8) versus 6.7 (3.9–11.0) for leukocytes and 2.2 (0.6–41.6) versus 2.1 (0.6–34.8) for CRP. Accordingly, there were no statistical changes of these parameters neither within nor between the groups.

## 4. Discussions

The main finding in this placebo-controlled patient blinded prospective study was that the immunomodulatory Agaricus *blazei* Murill-based mushroom extract AndoSan^TM^ [14] increasingly improved clinical symptoms in patients of both genders during a 3 weeks’ study period. In the placebo group there was an improvement of symptoms exclusively for women, although less than in the AndoSan^TM^ group and not significant. There were no significant differences between the study groups, but a close to significant p-value (0.054) when comparing the symptom score for men.

Of the four items (general well-being, abdominal pain, number of liquid stools per day, complications) of the SCDAI [26] there were improvements of key symptoms such as of number of liquid stools for both genders and pain for men. Interestingly, despite the trend in the AndoSan^TM^ group towards more stricturing disease (p = 0.04), less inflammatory disease (p = 0.04), significantly more ileal (p = 0.01) and ileocolic resections (p = 0.02) (Table 1), which implied increased disease activity vs. the placebo group, the improvement of symptoms as a whole was only evident in the former group.

Compared with the normal population the CD patients had considerably more fatigue, especially physical fatigue probably owing to the somatic manifestations of this chronic granulomatous disease. However, we were not able to demonstrate any advantage using AndoSan^TM^ vs. placebo on outcome of fatigue. At visit 3 there was as a whole an improvement in mental-, physical- and total fatigue in the placebo group vs. only the two latter fatigue scores in the AndoSan^TM^ group (Table 4). Thus, despite external factors that presumably could influence mental fatigue due to intercurrent subclinical and mental disease, we conclude that the mushroom extract vs. placebo had no demonstrable effect on fatigue in these patients.

For HRQoL in the AndoSan^TM^ group as a whole there was an improvement in bodily pain at visit 3, which is interesting because pain was the second item of the SCDAI that was significantly reduced for men at visit 3. Common to the AndoSan^TM^ and placebo groups were the improvements of vitality, whilst social functioning also was improved in the latter group. With the exception of improvement in bodily pain, which is a crucial factor in patients with CD suffering from intestinal and other symptoms, we conclude that the effect of AndoSan^TM^ on HRQoL was not different from placebo. Besides a reduction of bilirubin in the AndoSan^TM^ group and an increase of thrombocytes in the placebo group there was as reported [22] in a one-armed pilot study with 12 days’ AndoSan^TM^ consumption in CD patients, no alterations in general blood parameters including CRP as well as fecal calprotectin.

In a recent similar placebo-controlled study [23] of patients with UC consuming AndoSan^TM^, the improvements in addition to symptoms irrefutably also were evident for fatigue and HRQoL. Obviously, clinical improvement due to intake of this mushroom extract in patients with CD was more limited vs. those with UC. This may partly be explained by the fact that CD is pan-intestinal and characterized by transmural inflammation complicated by stenosis and/or development of fistulas in addition to more systemic manifestations (e.g. joints and anal fissures).

The AndoSan^TM^ and placebo groups had similar baseline values with respect to symptom score, fatigue and quality of life. The groups were also quite comparable with regard to disease duration, arthralgia and relevant comorbidity. However, there were significantly more resections in the AndoSan^TM^ group (20 vs. 9) and, accordingly, more stricturing and severe disease. Analyses of symptom score and fecal calprotectin were similar when we did calculations comparing the patients with inflammatory and stenotic presentation (data not shown).

The patients did not discontinue consumption of the mushroom extract throughout the study, since there were no side effects and normal blood samples, including liver function. As recently outlined [30], AndoSan^TM^ and AbM *per se* have been well tolerated by the patients in several clinical studies for hepatitis B [31] and C [30] and different cancers (breast, ovarian, myeloma) [32–34]. AbM induced temporary urticaria and moderate liver dysfunction developed after two months of AbM consumption [33], in one out of 78 patients only, which also received chemotherapy for ovarian cancer. With our knowledge of AndoSan^TM^ and AbM, and review of the literature, we believe it is safe as a supplement to patients with different types of disease.

The patients were not blinded for the authors leaving possible bias with respect to different attitude towards patients in the two groups. This is especially true for the first author who was responsible for the inclusion and randomization of participants, the implementation of the practical aspects of and in meeting with the patients, and also in the analysis of the results. This is a relatively small study, although with some significant results, with its limitations. Reduced compliance in carrying out the study, with missing or incorrect oral intake of AndoSan^TM^ or placebo, may be a possible source of error, even though this was not the impression in conversation with patients during and after the study period of three weeks.

Regarding possible drug interactions, AndoSan^TM^ did less than green tea inhibit the transmembrane efflux P-gp pump present in intestines and liver and hence important for drug absorption and excretion [35]. However, possible interactions may occur with P-gp substrates, e.g. some anti-cancer, diarrhea (loperamide) and cardiac (digoxin) agents, and P-gp inhibitors, e.g. verapamil. When testing AndoSan^TM^ on cytochrome P-450 metabolism, the extract inhibited it, but far less than green tea and clinically relevant systemic interactions were therefore considered unlikely [36]. In our clinical study, none of the CD patients were concomitantly treated with the above-mentioned anticancer-, heart-, or diarrhea drugs.

There are several studies of *Agaricus blazei* Murill, *Hericium erinaceus* and *Grifola frondosa*, isolated or in the mixture together as AndoSan^TM^, showing beneficial immunomodulatory, antioxidant, antihyperglycemic and anti-tumor effects [13, 14, 37–39]. However, most data, to date, were produced using rodents or cell cultures, and there are a very limited number of studies measuring their effects in humans.

In a placebo-controlled study in patients with gynecological cancer, AbM treatment in addition to chemotherapy was reported to increase NK cell activity in blood and improved the patients quality of life [40].

A randomized double-blind placebo-controlled study on 30 critically ill ICU patients, followed for 7 days, found significant increases in NK cell activities when given immune-enhancing enteral nutrition (IMHP) enriched with ß-glucan from the baseline and significantly greater increase than the control group [41]. The authors suggest that ß-glucan can be an attractive candidate to add to IMPH for stimulation of protective immunity without enhanced inflammation in critically ill patients. This finding is in line with previous studies in which ß-glucan enhanced NK cell activation in mice [42, 43].

A randomized double-blinded clinical trial in 57 elderly females found no immunomodulatory effect as measured by unaltered plasma levels of IL-6, TNFα and IFNγ after ingesting dried AbM capsules for a 60-day study period [44].

The notion of a potential anti-inflammatory effect of AndoSan^TM^ intake was a result of the surprising finding of reduced serum pro-inflammatory cytokines (IL-1ß, IL-6, IL-8) and chemokines (MCP-1, G-CSF, GM-CSF) in a pilot safety study with AndoSan^TM^ in healthy individuals without a placebo control [24].

In a study on healthy volunteers ingesting AndoSan^TM^ [37] there was a reduction *in vivo* of ROS mainly reflecting superoxide ions, and again pointing to an anti-inflammatory effect. However, this result was not demonstrated in the CD patients in the aforementioned pilot study (data not shown). The reason for reduced superoxide anions may be related to reduction of IL-1ß because inhibitors of ROS reduce synthesis of this cytokine in macrophages [45].

Oral administration of AndoSan^TM^ is associated with low bioavailability due to polysaccharides, like ß-glucan, that is normally not taken up from the GI-tract as it is a non-degradable cellulose (the human tract normally only takes up monosaccharides). However, Rice et al [46] showed that soluble ß-glucans are able to bind directly and undergo internalization by intestinal epithelial cells and gut associated lymphoid tissue (GALT) cells. The internalization of soluble ß-glucan by intestinal epithelial cells is not dependent on dectin-1, however in GALT cells dectin-1 and TLR-2 participate in uptake of soluble ß-glucan.

Recently, a steroid 4-hydroxy-17-methylincisterol (4-HM) [12] isolated from AbM dose-dependently suppressed the synthesis in PHA-stimulated peripheral blood mononuclear cells of cytokines IL-2, IL-4, IFNγ and TNFα by decreasing both NF-AT (nuclear factor of activated T-cells), which belongs to a family of transcription factors required for activation and proliferation of T lymphocytes including production of the first three aforementioned cytokines, and NF-κB—the latter being the “mother” of all inflammation.

Alkaline and aqueous substances isolated from AbM [11] had, when given orally to rats for 1–2 weeks, several anti-inflammatory effects such as improved healing of stress-induced ulcers and reductions of paw edema in the presence of nystatin or Freund’s adjuvant, as well as reduced neutrophil migration to the peritoneal cavity. It was speculated that these effects in part were related to modulations of cytokine levels for TNFα and IL-8. In another recent study [47] a water soluble polysaccharide isolated from AbM was given orally for 8 weeks to ovarectomized and osteopenic rats, and it markedly decreased serum levels of IL-1β, TNFα, ICAM-1 and total antioxidant status. AbM contains absorbable low-molecular weight anti-oxidant substances [10] that down-regulate the levels of reactive oxygen species (ROS) *in vitro*. Type 1 diabetes has similarities to CD because it has been regarded both as an autoimmune and as an innate inflammatory disease affecting the pancreas [48]. Anecdotes of possible benefits of AbM for diabetes in folk medicine have been supported by findings in a rodent model for diabetes [9]. Since the hypoglycemic effect of AbM seems to result from its suppression of oxidative stress and pro-inflammatory cytokine production [38], similar therapeutically mechanisms probably play a role in CD patients who responded positively on the AndoSan^TM^ treatment in the current study.

There also was an anti-allergic effect in mice sensitized to ovalbumin (OVA), as demonstrated by reduction of specific anti-OVA IgE antibodies, both when AndoSan^TM^ was given before or after the OVA immunization [49]. In this allergy model also there was an increase in Th1 relative to Th2 cytokines in spleen cell cultures *ex vivo* obtained from the animals treated with AndoSan^TM^ [49]. In addition, the inhibitory effect of an isolated carbohydrate fraction of AndoSan^TM^ [15] on the tissue degrading pro-inflammatory and tumor-associated enzyme, legumain (aspariginyl endopeptidase), that probably may also contribute to less inflammatory activity in the CD patients.

Pharmaceutical investigation of AndoSan^TM^ revealed the carbohydrate content to be only 2% of the ~5mg dry material/ml, and that it was concentrated in the polar high molecular weight fraction of AndoSan^TM^ [15]. It was this fraction that was the most potent inhibitor of legumain [15]. The mushroom extract also contains some not yet characterized proteins (personal communication, prof G Vegarud at The Norwegian University of Life Sciences). As to the pharmacokinetics of AndoSan^TM^ that is a mixed water extract of mycelium to the aforementioned three *Basidiomycetes* mushrooms, it has biological effects when taken orally both in mice and men. Ingredients in AndoSan^TM^ may execute their effects directly after absorption through intestinal mucosa enterocytes to the blood and processing in the liver, where Kuppfer cells and endothelial cells may be stimulated similar to our previous findings in monocyte/macrophage and endothelial cell cultures [50, 51]. However, substances in the mushroom extract may have a greater indirect influence on the body by inducing changes in the microbiota and production of analytes by bacteria after their uptake of sugar moieties for energy etc.. Moreover, substances such as β-glucan my further be transported by dendritic cells to lymphocytes in GALT and induce local immune responses there, or systemic if circulated in blood [52, 53].

In conclusion, we found increasingly improved symptom score in the AndoSan^TM^ group, of both genders, foremost regarding stool frequency and abdominal pain. Compared with placebo there was a significant reduction in stool frequency, favoring the AndoSan^TM^ group. For fatigue, quality of life, calprotectin in feces and blood samples there were comparable results between the study groups. We suggest that AndoSan^TM^ may be used as a safe supplement to conventional medication to relive symptoms in these patients. At present, the effects on cytokine levels from AndoSan^TM^ consumption in these patients are being studied.

## Supporting Information

S1 Table CONSORT 2010 Checklist. (DOC)

S1 Text Study Protocol Norwegian version. (DOCX)

S2 Text Study Protocol English version. (DOCX)
